# The burden of RSV-associated illness in children aged < 5 years, South Africa, 2011 to 2016

**DOI:** 10.1186/s12916-023-02853-3

**Published:** 2023-04-11

**Authors:** Jocelyn Moyes, Stefano Tempia, Sibongile Walaza, Meredith L. McMorrow, Florette Treurnicht, Nicole Wolter, Anne von Gottberg, Kathleen Kahn, Adam L. Cohen, Halima Dawood, Ebrahim Variava, Cheryl Cohen

**Affiliations:** 1 Center for Respiratory Diseases and Meningitis, National Institute for Communicable Diseases of the National Health Laboratory Service, Private Bag X4, Sandringham, 2131 Johannesburg, Gauteng South Africa; 2 School of Public Health, Faculty of Health Sciences, University of the Witwatersrand, Johannesburg, South Africa; 3 Division of Viral Diseases, Centers for Disease Control and Prevention, Atlanta, GA USA; 4 Division of Virology, Faculty of Health Sciences, National Health Laboratory Service, Charlotte Maxeke Johannesburg Academic Hospital, Johannesburg, South Africa; 5 School of Pathology, Faculty of Health Sciences, University of the Witwatersrand, Johannesburg, South Africa; 6 MRC/Wits Rural Public Health and Health Transitions Research Unit (Agincourt), School of Public Health, Faculty of Health Sciences, University of the Witwatersrand, Johannesburg, Epidemiology and Global Health Unit, Johannesburg, South Africa; 7 Influenza Division, Centers for Disease Control and Prevention, Atlanta, GA USA; 8 Influenza Program, Centers for Disease Control and Prevention, Pretoria, South Africa; 9 Department of Medicine, Pietermaritzburg Metropolitan Hospital, Pietermaritzburg, South Africa; 10 Caprisa, University of KwaZulu-Natal, Pietermaritzburg, South Africa; 11 Department of Medicine, Klerksdorp-Tshepong Hospital Complex, Klerksdorp, South Africa; 12 Department of Medicine, Faculty of Health Sciences, University of the Witwatersrand, Johannesburg, South Africa; 13 Perinatal HIV Research Unit, University of the Witwatersrand, Johannesburg, South Africa

**Keywords:** Burden, Respiratory syncytial virus, Children, Respiratory illness

## Abstract

**Background:**

Vaccines and monoclonal antibodies to protect the very young infant against the respiratory syncytial virus (RSV)-associated illness are effective for limited time periods. We aimed to estimate age-specific burden to guide implementation strategies and cost-effectiveness analyses.

**Methods:**

We combined case-based surveillance and ecological data to generate a national estimate of the burden of RSV-associated acute respiratory illness (ARI) and severe acute respiratory illness (SARI) in South African children aged < 5 years (2011–2016), including adjustment for attributable fraction. We estimated the RSV burden by month of life in the < 1-year age group, by 3-month intervals until 2 years, and then 12 monthly intervals to < 5 years for medically and non-medically attended illness.

**Results:**

We estimated a mean annual total (medically and non-medically attended) of 264,112 (95% confidence interval (CI) 134,357–437,187) cases of RSV-associated ARI and 96,220 (95% CI 66,470–132,844) cases of RSV-associated SARI (4.7% and 1.7% of the population aged < 5 years, respectively). RSV-associated ARI incidence was highest in 2-month-old infants (18,361/100,000 population, 95% CI 9336–28,466). The highest incidence of RSV-associated SARI was in the < 1-month age group 14,674/100,000 (95% CI 11,175–19,645). RSV-associated deaths were highest in the first and second month of life (110.8 (95% CI 74.8–144.5) and 111.3 (86.0–135.8), respectively).

**Conclusions:**

Due to the high burden of RSV-associated illness, specifically SARI cases in young infants, maternal vaccination and monoclonal antibody products delivered at birth could prevent significant RSV-associated disease burden.

**Supplementary Information:**

The online version contains supplementary material available at 10.1186/s12916-023-02853-3.

## Background

Globally, an estimated 3.3 million RSV-associated lower respiratory tract infection (LRTI) hospitalizations and between 26,300 and 101,400 deaths occur annually in children aged < 5 years. A large proportion of these hospitalizations and deaths occur in low and middle-income countries (LMIC) [1].

New RSV prevention technologies in the pipeline include maternal vaccination and long-acting monoclonal antibodies (MAB) [2]. Describing the burden of RSV-associated mild and severe disease is pivotal for estimating the cost-effectiveness of these new interventions. Although incidence data of hospitalization with RSV-associated LRTI from South Africa have been published these data did not include national estimates and did not quantify non-medically attended disease burden nor mild illness associated with RSV [3]. Burden estimates in fine age groups (in infants < 1 year) including non-medically attended illness will also be used to improve the parameterizing of cost-effectiveness models, which will be valuable for policy makers.

Additional burden of disease may lie in mild illness, and therefore leaving this group out of estimates may significantly underestimate the burden, cost, and cost-effectiveness of interventions [4]. Due to polymerase chain reaction (PCR) testing the identification of viruses in the respiratory tract has become more sensitive, the presence of these viruses may not be associated with disease, therefore adjusting for the attributable fraction of RSV strengthens burden estimates (paper submitted to the journal of infection). The burden of non-medically attended RSV-associated illness has not been estimated in our setting; however, estimates for influenza suggest that there may be a significant burden of disease in non-medically attended illness [5]. Data published in our setting, model out of hospital death in the < 5 years age group, refining these estimates to finer age bands will improve mortality burden estimates [6]. Describing the seasonality of RSV may assist with the implementation of RSV prevention technologies by targeting immunizations and MAB administration prior to and during the peak RSV transmission season [2].

We aim to describe the full burden of RSV-associated illness in South African children aged < 5 years (both medically and non-medically attended illness) in 1-month age groups for infants and 3-month age groups until 2 years and then yearly until < 5 years; specifically, we describe the burden of RSV-associated acute respiratory illness (ARI), severe acute respiratory infection (SARI) and mortality (in- and out-of-hospital) in South Africa during 2011 to 2016.

## Methods

We estimated the burden of medically attended and non-medically attended RSV-associated acute respiratory illness (ARI) and severe acute respiratory illness (SARI) in children aged < 5 years by using a previously described approach [5, 7, 8]. We estimated the burden of RSV-associated illness for six defined endpoints.

We estimated burden in the following categories:

1. *Medically attended acute respiratory illness (ARI)*: a child with ARI who sought medical care with a health care provider
2. *Non-medically attended ARI*: a child with ARI who did not present to any healthcare provider excluding pharmacies.
3. *Medically attended SARI*: a child hospitalized with physician-diagnosed LRTI.
4. *Non-medically attended SARI*: a child with LRTI who did not present to any healthcare provider excluding pharmacies.
5. *Medically attended RSV-associated death*: a child who died in hospital following admission with RSV-associated SARI.
6. *Non-medically attended RSV-associated death*: a child who was considered to have died of RSV-associated Illness outside of a hospital

We refer to ARI as a mild illness and SARI as a severe illness in the remaining text. We also refer to the medically attended illness as MA and non-medically attended illness at NMA.

### Data sources

#### National sentinel surveillance program (NSP) for severe respiratory illness

Since 2009 the National Institute for Communicable Diseases (NICD), South Africa, has conducted prospective systematic hospital-based sentinel surveillance for severe respiratory illness in five of the nine South African provinces. In 2012, outpatient surveillance was added at clinics in the catchment area of two sentinel hospitals. Sentinel site data from the Edendale Hospital (and Gateway Clinic) in KwaZulu-Natal Province (KZN), and the Klerksdorp-Tshepong Hospital Complex (KTHC) (and Jouberton Clinic), in the North West Province (NWP) supply data for the base provincial estimates [9]. Children were enrolled into the surveillance system based on the following case definitions: hospitalized children were enrolled based on physician-diagnosed LRTI and those enrolled from the clinic were enrolled on the ILI case definition of cough and fever (measured or reported) with a duration of < 10 days. We obtained data from the NSP for the observed number of severe cases at base hospitals and the RSV-associated detection rates for mild and severe illnesses. Additional details of the surveillance methodology are available in Additional file 1.

#### National census data and Demographic Health Survey (DHS)

Population denominators (for hospital catchment areas and provincial populations) were obtained from the National Census of 2011, annual adjustments were made to these estimates using regional and national adjustment factors supplied by Statistics South Africa [10–12]. Population data are supplied in one-year age bands, therefore in the < 1-year group, an adjustment factor was applied to obtain numbers in fine age strata. This was calculated by adjusting the < 1-year population for neonatal and infant mortality rates to allocate population by month of life. Data for neonatal and infant mortality were obtained from (DHS) [3]. The DHS and published data were used for the prevalence and relative risk estimates for pneumonia risk factors [13, 14]. These data were used to adjust the provincial severe illness estimates in non-base provinces (as described in the analysis section and Additional file 1) including HIV infection, exposure to indoor air pollution, crowding, malnutrition, low birth weight, and non-exclusive breastfeeding.

#### Health care Utilization Surveys (HUS)

We conducted HUS in 3 provinces to estimate the proportion of mild and severe respiratory illness that was non-medically attended [15, 16]. The HUS also provided estimates of health-seeking behavior, to adjust for cases (mild and severe) that did not seek care at the base sentinel sites.

#### Published data

We made use of published attributable fraction estimates derived from comparing the prevalence of RSV in cases to healthy controls in South Africa to adjust burden estimates for the attributable fractions of RSV illness [17]. We also used published data to adjust our observed ILI estimates for the proportion of children who present without fever [18].

### Data analysis

#### Estimating the number and incidence rate of RSV-associated MA severe illness

We used the methodology described by Fuller et al. to estimate the national mean annual burden of MA RSV-associated severe illness from a base hospital surveillance site with a defined catchment population [9]. With this method, the rate of severe illness at this base site is then applied to a base provincial (or similarly defined geographic area with defined populations) population, thereby providing a rate of severe illness in that province. To extrapolate from this base provincial rate to all other provinces in the country, adjustments are made for the prevalence of risk factors for severe respiratory illness in the other provinces. Provincial rates are then applied to national population numbers to estimate a national burden rate/100,000 population. This methodology is further described in the following four steps. All formulae used are presented in Additional file 1.

##### Step 1: Estimating MA severe illness rates in base provinces (NWP and KZN)

We estimated severe illness hospitalization rates for Edendale (KZN) and KTHC (NWP) sentinel surveillance hospitals, these were adjusted for non-enrolment due to weekends, refusals/non-enrolment on surveillance days, and health-seeking behavior [15, 16]. The population denominators for the hospital catchment area were derived from census projections and rates of severe illness in the catchment area were applied to provincial population denominators. The formula for estimation of the adjusted rate of severe illness in the base provinces is described in Additional file 1.

##### Step 2: Estimate the MA severe illness rate in the other 7 provinces

To estimate the MA severe illness rates in the other 7 provinces the base provinces (NWP and KZN) severe illness rates were adjusted for provincial-level prevalence of risk factors for pneumonia (including HIV infection, exposure to indoor air pollution, crowding, malnutrition, low birth weight, and non-exclusive breastfeeding) for each of province [19].

##### Step 3: Estimation of rates of MA RSV-associated severe illness in all provinces

The provincial rates of MA RSV-associated severe illness were calculated by multiplying the provincial severe illness rates by the MA RSV detection rates (number of RSV-positive severe illness cases/number of severe illness tested) at sentinel surveillance sites (all sentinel sites) and adjusted by the attributable fraction of RSV. The attributable fraction of RSV was obtained from a published study [17].

##### Step 4: Estimation of the number and incidence rate per 100,000 populations of MA RSV-associated severe illnesses in all provinces

We estimated the provincial number of RSV-associated MA severe illnesses by multiplying the provincial RSV-associated MA severe illness rates by the population at risk in each province over the study period. Provincial estimates are added together to obtain a national estimate. National rates are estimated by dividing total RSV-associated severe illness episodes by the national population and presented per 100,000 persons (Additional file 1).

#### Estimating the number and incidence rate of NMA RSV-associated severe illness

The same methodology was applied as for MA RSV-associated severe illness making use of NMA attended numbers and rates, we used the following four steps: (i) estimating the NMA severe illness rates in the base province by adjusting the MA severe illness rates by the proportions of individuals who did not seek care for the symptoms of severe illness [15, 16]. (ii) Estimate the NMA severe illness rates in other provinces applying the same adjustments for the risk factors for pneumonia as for medically attended severe illness obtained from the DHS. (iii) Estimate the NMA RSV-associated severe illness rate by applying the RSV-detection rate from our surveillance data, and (iv) estimate the national NMA attended a number of RSV-associated severe illnesses by multiplying provincial rates by provincial population estimates and then sum the provincial estimates. National rates are estimated by dividing total NMA RSV-associated severe illness episodes by the national population and presented per 100,000 persons (Additional file 1).

#### Estimating the number and incidence rate of MA RSV-associated mild illness

Surveillance for mild illness is only conducted at one of several clinics serving each of the base hospital catchment areas using the case definition of ILI. The base ILI rates of illness are derived by a backward adjustment of the severe illness rate at sentinel hospitals, adjusting for mild illness cases transferred to the hospital and in-referrals for other facilities (Additional file 1). We use mild illness rates at sentinel sites as proxies for the base provinces and expand to other provinces and nationally in a similar way to the severe illness and RSV-associated severe illness estimates but without adjusting for the severe illness risk factors. As RSV-associated illness may frequently present without fever we adjusted our ILI estimates by multiplying ILI estimates by an adjustment factor for the proportion of ARI without fever [20]. This provides the ARI estimate (mild illness).

#### NMA mild illness

We estimated the NMA mild illness we used a similar method to the estimates for MA mild illness but adjusted for the proportion who did not seek health care for mild illness from the HUS [15, 16].

#### Mortality estimates

In-hospital mortality was estimated by applying the age-specific observed (NSP) RSV-associated case-fatality ratio to the national number of medically attended RSV-associated severe illnesses episodes in this burden analysis. To estimate out-of-hospital mortality we used published data on the proportion of deaths that occurred out-of-hospital. In-hospital deaths were adjusted for this proportion to give the total estimated number of deaths [21].

Confidence intervals (CI) for medically and non-medically attended RSV-associated illness are obtained by using bootstrapping resamples over 100 replications for all parameters. The lower limit and upper limit of the CI are the 2.5th and 97.5th percentiles of the estimated values from 1000 resampled datasets.

#### Seasonality

The seasonality of RSV-associated illness was derived from the NSP mentioned above. The weekly number of positive hospitalized RSV cases is divided by the total number of cases to define the weekly detection rate of RSV. The detection rate is plotted by epidemiologic week for each year of surveillance. A mean weekly detection rate over 5 years is used to define the average seasonality when the detection rate rises above 10% and is maintained for at least two weeks.

## Results

### National burden of RSV-associated illness (MA and NMA)

The mean annual number of RSV-associated mild illness cases in children aged < 5 years was 264,112 (95% confidence interval (CI) 134,357–437,187); of these 36% (96,783) were in children aged < 1 year (Additional file 1: Table S1).

The mean annual number of RSV-associated severe illness cases in children aged < 5 years was 96,220 (95% CI 66,470–132,844). Eighty-two percent of these, 78,571 (95% CI 56,187–105,831) were in children aged < 1 year. Infants younger than < 3 months of age accounted for 40% (27,982/78,571) of the burden in this age group (Additional file 1: Table S2).

### National burden of RSV-associated mild illness

The highest burden of RSV-associated mild illness was in the 2-month age group with a total of 17,259 (95% CI 8776–26,759) cases or 18,361/100,000 population (95% CI 9336–28,466). This decreases to 4571/100,000 (95% CI 19,622–81,555) by 10 months but increases again to 11,935/100,000 (95% CI 6952–18,025) in the 12–14-month age group and to 10,261 (95% CI 4305–17,507) in the 21–23-month age group. (Additional file 1: Table S1).

**Fig. 1:**
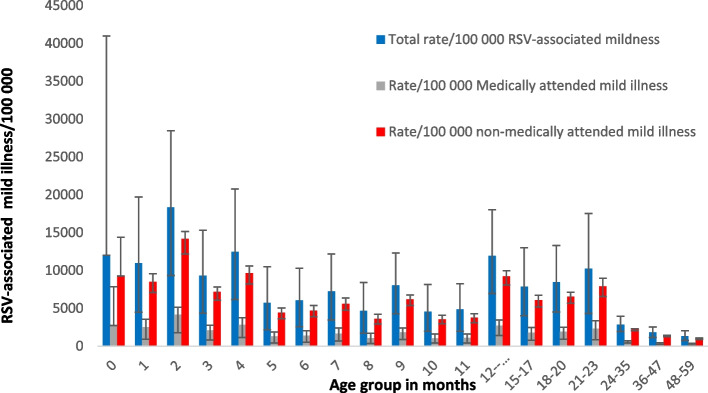
Estimated incidence rates of RSV-associated mild illness, medically and non-medically attended, per 100,000 population, by age group, South Africa, 2011–2016. Numbers and confidence intervals are documented in Additional file 1: Table S1. Error bars indicate 95% confidence intervals

The rate/100,000 for mild cases drop substantially from the age group 12–14- to the 48–52-month age group (14,755 (95% CI 1014–2114) and 127 (95% CI 59–214), respectively). The burden of mild illness is higher in NMA cases in all age groups (Fig. 1).

### National burden of RSV-associated severe illness

**Fig. 2:**
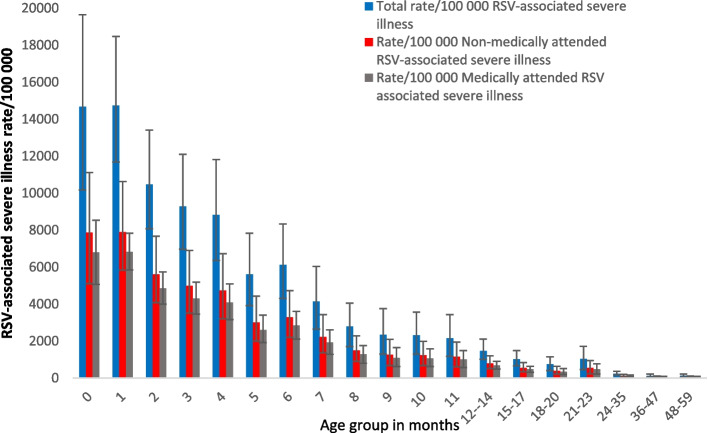
Estimated incidence rates of RSV-associated severe illness, medically and non-medically attended, per 100,000 population, by age group, South Africa, 2011–2016. Numbers and confidence intervals are described in Additional file 1: Table S2. Error bars indicate 95% confidence intervals

The mean annual number of cases of severe illness was 96,220 cases (95% CI 66,470–132,844) annually in South Africa, of which 78,571 (95% CI 56,187–105, 831) were in infants. The highest burden of RSV-associated severe illness was in the < 1-month age group, with 14,110 (95% CI 9784–18,889) cases nationally (Fig. 2). Thirty-six percent of cases in children aged < 1 year are in the < 1 month and 1-month age groups (27,982/78,571). The rate per 100,000 was similar in the < 1 month and 1-month age groups (14,674 95% CI 10,175–19,645 vs 14,736 95% CI 11,689–18,472). The rate of RSV-associated severe illness decreased to 2156/100,000 (95% CI 1192–3439) by 11 months. MA cases accounted for 46% (44,615/96,220) of severe illness cases in children aged < 5 years (Additional file 1: Table S2).

More than half (54%) of the cases of severe illness are NMA in the < 1-year age group (42,143/78,571).

### RSV-associated mortality

The mean annual number of RSV-associated deaths was 651 (95% CI 445–861) cases in children < 5 years. The rate of RSV-associated deaths was highest in the first and second month of life 110.8 (95% CI 74.8–144.5) and 111.3 (95% CI 86.0–135.8), respectively. Out-of-hospital deaths accounted for 26% of the total deaths in children aged < 5 years, with 154 (95% CI 107–207) deaths occurring in infants and 161 (95% CI 115–227) deaths in children aged < 5 years (Table 1).

**Table 1:** Estimated mean annual number and rates of respiratory syncytial virus-associated deaths in children aged < 5 years, South Africa. 2011–2016

Age group: months	Total		Medically attended deaths^a^		Non-medically attended deaths^b^	
	**Number (95% CI)**	**Rate**^**c**^** (95% CI)**	**Number (95% CI)**	**Rate**^**c**^** (95% CI)**	**Number (95% CI)**	**Rate**^**c**^** (95% CI)**
< 1	109 (73–142)	110.8 (74.8–144.5)	81 (56–108)	82.0 (56.9–109.8)	28 (18–34)	28.8 (18.0–34.7)
1	107 (83–131)	111.3 (86.0–135.8)	79 (63–99)	82.4 (65.3–103.2)	28 (20–31)	28.9 (20.6–32.6)
2	76 (57–95)	79.1 (59.5–98.7)	56 (43–72)	58.6 (45.2–75.0)	20 (14–23)	20.6 (14.3–23.7)
3	67 (49–85)	70.2 (51.3–89.0)	50 (37–65)	51.9 (39.0–67.6)	17 (12–20)	18.2 (12.3–21.4)
4	64 (45–83)	66.6 (46.8–86.9)	47 (34–63)	49.3 (35.6–66.1)	17 (11–20)	17.3 (11.2–20.9)
5	40 (27–55)	42.4 (28.8–57.6)	30 (21–42)	31.3 (21.9–43.8)	11 (7–12)	11.0 (6.9–13.8)
6	40 (27–53)	41.6 (28.6–55.2)	29 (21–40)	30.8 (21.7–41.9)	10 (7–13)	10.8 (6.9–13.2)
7	27 (17–38)	28.1 (17.6–39.9)	20 (13–29)	20.8 (13.3–30.3)	7 (4–9)	7.3 (4.2–9.6)
8	18 (11–26)	18.9 (11.3–26.8)	13 (8–19)	14.0 (8.6–20.4)	5 (3–6)	4.9 (2.7–6.4)
9	15 (8–24)	16.0 (8.6–24.8)	11 (6–18)	11.8 (6.5–18.9)	4 (2–6)	4.2 (2.1–6.0)
10	15 (8–22)	15.7 (8.6–23.6)	11 (6–17)	11.6 (6.6–17.9)	4 (2–5)	4.1 (2.1–5.7)
11	14 (7–21)	14.6 (7.9–22.7)	10 (6–16)	10.8 (6.0–17.2)	4 (2–5)	3.8 (1.9–5.4)
12–14	14 (9–19)	4.8(3.2–6.7)	10(7–14)	3.6 (2.5–5.1)	4 (2–5)	1.3 (0.8–1.6)
15–17	10 (6–13)	3.4 (2.1–3.7)	7 (5–10)	2.5 (1.6–3.6)	2 (1–3)	0.9 (0.5–1.1)
18–20	7 (4–10)	2.5 (1.3–5.5)	5(3–8)	1.8 (1.0–2.8)	2 (1–3)	0.6 (0.3–0.9)
21–23	10 (4–15)	3.4 (1.5–5.5)	7 (3–12)	2.5 (1.2–4.2)	2 (1–4)	0.9 (0.4–1.3)
24–35	9 (6–13)	0.8 (0.5–1.2)	7 (5–10)	0.6 (0.4–0.9)	2 (1–3)	0.2 (0.1–0.3)
36–47	4 (2–7)	0.4 (0.2–0.7)	3 (2–6)	0.3 (0.1–0.5)	1 (0–2)	0.1 (0.0–0.2)
48–59	5 (2–7)	0.4 (0.2–0.7)	3 (2–6)	0.3 (0.1–0.5)	1(0–2)	0.1 (0.0–0.2)
< 1 year	592 (412–777)	55.5 (38.9–75.0)	438 (339–654)	38.1 (29.5–57.0)	154 (107–207)	13.4 (9.3–18.0)
< 5 years	651 (445–861)	11.6 (8.6–16.9)	490 (364–720)	8.6 (6.5–12.9	161 (115–227)	3.0 (2.1–4.1)

*Abbreviations*: *CI* Confidence intervals

^a^Medically attended deaths defined as occurring in-hospital

^b^Non-medically attended deaths defined as occurring out-of-hospital

^c^Rates expressed per 100,000 population

### Seasonality of RSV

**Fig. 3:**
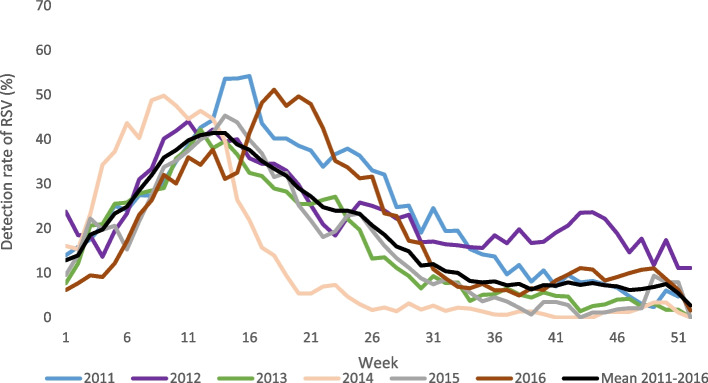
Weekly detection rate of RSV-associated severe illness 2011–2016, in children aged < 5 years, South Africa, 2011–2016. Data are obtained from sentinel surveillance; weekly number of RSV-positive cases (hospitalized children) < 5 years divided by the total number of cases enrolled into surveillance. The graph depicts a 2-week rolling average

Although RSV is detected throughout the year in South Africa, an annual peak of RSV circulation is observed in the months of April and May. The mean peak of RSV circulation (2011–2016) was week 14 (second week of April), with a mean peak detection rate of RSV in children hospitalized with LRTI of 42% (range 39% to 65%) (Fig. 3).

## Discussion

We were able to document the national burden of RSV-associated illness in young infants by month of life for both mild and severe RSV-associated illness in a LMIC, specifically demonstrating the highest burden of illness is in the first and second month of life. This analysis also describes the significant burden of non-medially attended RSV-associated illness and is the first to document this burden on our setting. This more inclusive description of burden supports interventions such as maternal vaccination and long-acting MABs which target the younger infant. Our analysis adds methodologic techniques that provide more useful data on the burden of RSV in South Africa: estimating medically and non-medically attended illness in each of these narrow age bands, adjusting estimates for RSV cases without fever, and using RSV-AFs to adjust the observed proportion of cases for a more robust estimate.

The burden of disease for mild illness is highest in the 2-month age group and the burden of NMA mild illness is higher than MA mild illness in all age groups. This likely reflects health-seeking behavior and access to primary health care in our setting, where many people may not seek care if illness is perceived as mild even though healthcare is free to children < 5 years. This likely reflects the health-seeking behavior and access to primary health care in our setting, where healthcare is free to children < 5 years. It also highlights the high burden of mild illness in communities and may support an intervention for older children. We adjusted RSV-associated ILI for the proportion of children who present with RSV-associated illness without fever. Nyiro et al. [18, 20] describe that up to 25% of children with RSV-associated mild illness present without fever. The adjustment assists in describing the substantial burden of RSV-associated in young children. While several estimates of RSV-associated mild or outpatient illness have been published from LMIC, few provide data in fine age bands. In Kenya, a similar methodology to ours Emukule et al. [4] described the outpatient burden of RSV-associated ARI including an estimate of non-medically attended RSV. The incidence of NMA RSV-associated ILI (6.0/1000 (95% CI 5.4–6.5) in their < 5-year age group was higher compared to 4.7/1000 (95% CI 3.3–66) for medically attended ILI, these estimates are similar to our estimates in this broad age group (< 5 years) but do not allow for comparison in finer age band [4]. This higher proportion of MA illness in our setting, may be due to cost-free access to care for children.

The burden of RSV-associated severe illness is highest in the first month and second month of life after which the burden declines to 11 months of life and further through years 2 to 4 of life. Young age is a well-described risk factor for RSV-associated severe illness and infants < 6 months will be the group of infants that will most benefit from prevention interventions (maternal vaccine of MAB) [22]. Although the burden of NMA severe illness is higher than MA severe illness there is still a significant burden of illness in this group. The burden of NMA attended severe illness may be due to socioeconomic factors impeding access to care. Similar to the severe RSV-associated illness burden, mortality due to RSV-associated respiratory illness is highest in the first 3 months of life, declining steadily until 11 months of life, with few deaths in the older age groups. There are data on the burden of RSV-associated severe illness in other LMICs, but most of these are in wider age bands than what we present here. Li et al. [1] published a meta-analysis of global RSV-associated hospitalization incidence; these results reflect a large burden of disease in infants less than 4 months of age in LMIC countries (31/1000, 95% CI 17.0–56.4) and are similar to our estimates in the same age group. However, our data estimates the burden in < 1-month, < 2-month, and < 3-month age groups, illustrating how larger age bands dilute the substantial burden in the first two months of life. Our estimates are supported by an analysis done in South Africa which estimates the hospitalization rate for RSV-associated illness to be 7910/100,000 population (95% CI 6155–9665), very similar to our MA RSV-associated severe illness estimates of 6804/100,000 (95% CI 5065–8529) in the < 1 month of age and through the first year of life [3]. A cohort study from South Africa, reports incidence rates in the first 2 years of life (RSV-associated illness) of 0.07 cases per child year (95% CI 0.05–0.10) for hospitalized RSV-associated LRTI in children < 6 months. These estimates are very similar to the estimates in this study. With these comparable estimates in hand, we provide policy makers with evidence to consider RSV-prevention interventions to protect young infants in our setting [23]. In Kenya, using a similar methodology to ours, the burden of disease for severe cases included NMA severe RSV-associated illness in children aged < 1 year was 14.5/1000 (95% CI 8.9–23.7) which is lower than our estimates of 3752/100,000 (95% CI 2530–5357) [4]. However, our estimates for non-medically attended mild illness (6657/100,000 (95% CI 2692–12,540)) were higher than the estimates for mild non-medically attended illness in Kenya 8.9/1000 (95% CI 4.8–16.7) [4]. The difference may be due to different health-seeking behaviors in the different settings.

An additional strength of our study was the ability to adjust numbers by the AF-RSV, described in our setting [17]. This is important because the detection of respiratory viruses does not always reflect their role in causing disease [17, 24]. While the AF for RSV was generally high in infants, in children aged 1–4 years the AF-RSV was 74.6% (95% CI 53.6–86.0%) for mild illness and 83.4% (95% CI 70.9–90.5%) for severe illness. This adjustment refined our estimates in these age groups.

Although the case fatality ratio (CFR) for RSV-associated illness in infants (CFR between 1.8% (95% confidence interval (CI) 0.8–3.6%) and 1.0 (0.4–1.5) infants < 3 months and those 3–6 months respectively) is lower than other viral pathogens, such as influenza (CFR 3.2% (0.6–15.4%) [1, 25, 26]. Mortality in the young infants will increase years of life lost (YLL) and affect cost-effectiveness estimates of intervention to prevent RSV-associated severe illness. Out-of-hospital mortality is difficult to define, specifically where the cause of death and death registration is not documented. In an earlier analysis of excess mortality attributable to RSV, we estimated that approximately 26% of RSV-associated deaths in children aged < 5 years occur in the community [21].That model estimated 665 (95% CI 105–1105) deaths in children aged < 5 years to be RSV-associated very similar to this estimate of 650 (95% CI 479–947) deaths in children < 5 years. Shi et al. estimated that up to 49% of deaths from RSV-associated SARI, in children < 5 years, in LMIC countries occur out of hospital [27]. Other estimates from recent publications suggest that out-of-hospital deaths may account for a larger proportion of RSV deaths for example in India > 80% of RSV-associated deaths occur in the community, in Zambia the estimate was 62% and in Pakistan 27% [28–30]. There are many differences between these settings, including urban vs rural settings, cost of accessing health care, and healthcare system factors. Nonetheless, these newly published data suggest that our estimate may be a minimum estimate.

Variation in RSV seasonality by country, region, and climatic zone is well described, implying that the description of seasonality in each country is important [31, 32]. Describing country-specific seasonality will assist with decision-making regarding the seasonal vs all-year administration of interventions such as maternal vaccine and MAB treatment for young infants. Knowledge of RSV-seasonality will also provide important data for cost-effectiveness models.

Limitations of our study include: This method is sensitive to the description of the mild and severe cases in the base catchment area. Even though we made adjustment for non-enrolment, conducted a HUS and our base hospitals had defined catchment populations it is possible that cases were missed. While we were able to make use of observed data in many provinces (5/9), not all provinces of the country were included in the observed data, this may not account for the difference in the detection rate of RSV between provinces. The difference in the prevalence of risk factors from ecological data such as the DHS and published data may not account for all the differences between provinces. In the same way, health-seeking behavior may differ between rural and urban sites leading to over or underestimation of NMA mild and severe illness based on a HUS done at 3 sites. Population data were adjusted up from the 2011 census and may not accurately account for population movement and increases. Our analysis produced a wide confidence interval in some age groups, this is likely due to the low numbers in these finer age bands and the adjustment used from observed cases.

## Conclusions

The high burden of RSV-associated illness in children in South Africa, particularly very young infants, places a significant burden on healthcare systems. Interventions such as maternal vaccines and long-acting monoclonal antibodies may have a substantial impact on the burden to the healthcare system and the health of infants.

## Supplementary Information

**Additional file 1.** Contains information about the surveillance programme from which the observed data was drawn. This includes the laboratory methods used for testing of respiratory samples. Also included are the formulae used to calculate the SARI rates for the base provinces, SARI rate in other provinces, RSV-associated SARI rate in all provinces and SARI numbers in all provinces. Formulae are also included to the calculation of non-medically attended SARI and the burden of ARI. Each formula has a brief description of the inputs. The additional file 1 also contains two tables. Table S1: Estimated mean annual number and rates of respiratory syncytial virus-associated mild illness in children aged <5 years, South Africa, 2011-2016. This table documents the numbers used in the main manuscript figure 1 (burden by age group including medically and non-medically attended), similarly table S2: Estimated mean annual number and rates of respiratory syncytial virus-associated severe illness (excluding deaths) in children aged <5 years, South Africa, 2011-2016 documents the numbers (burden by age group, including medically and non-medically attended) used for the main manuscript figure 2.

## Data Availability

Data used for this manuscript is available through request to the first author, with approval from the surveillance program investigators.

## Abbreviations

AF: Attributable fraction
ARI: Acute respiratory illness
BREC: Biomedical Research Ethics Committe
CI: Confidence interval
DHS: Demographic and Health survey
HREC: Human Research Ethics Committee
HUS: Healthcare utilization survey
ILI: Influenza-like illness
KTHC: Klerksdorp Tshepong Hospital complex
KZN: Kwa-Zulu Natal
LMIC: Lower-middle income country
LRTI: Lower respiratory tract infection
MA: Medically attended
MAB: Monoclonal antibody
NICD: National Institute for Communicable Diseases
NMA: Non-Medically attended
NSP: National surveillance program
NWP: North West Province
PCR: Polymerase chain reaction
RSV: Respiratory syncytial virus
SARI: Severe acute respiratory illness
